# The Future of Cardiovascular Stents: Bioresorbable and Integrated Biosensor Technology

**DOI:** 10.1002/advs.201900856

**Published:** 2019-08-19

**Authors:** Daniel Hoare, Anubhav Bussooa, Steven Neale, Nosrat Mirzai, John Mercer

**Affiliations:** ^1^ BHF Cardiovascular Research Centre University of Glasgow G12 8TA Glasgow Scotland; ^2^ James Watt South Building School of Engineering University of Glasgow G12 8QQ Glasgow Scotland; ^3^ Bioelectronics Unit College of Medical, Veterinary & Life Sciences (MVLS) University of Glasgow G12 8QQ Glasgow Scotland

**Keywords:** atherosclerosis, bioresorbable, cardiovascular disease, sensors, smart stents

## Abstract

Cardiovascular disease is the greatest cause of death worldwide. Atherosclerosis is the underlying pathology responsible for two thirds of these deaths. It is the age‐dependent process of “furring of the arteries.” In many scenarios the disease is caused by poor diet, high blood pressure, and genetic risk factors, and is exacerbated by obesity, diabetes, and sedentary lifestyle. Current pharmacological anti‐atherosclerotic modalities still fail to control the disease and improvements in clinical interventions are urgently required. Blocked atherosclerotic arteries are routinely treated in hospitals with an expandable metal stent. However, stented vessels are often silently re‐blocked by developing “in‐stent restenosis,” a wound response, in which the vessel's lumen renarrows by excess proliferation of vascular smooth muscle cells, termed hyperplasia. Herein, the current stent technology and the future of biosensing devices to overcome in‐stent restenosis are reviewed. Second, with advances in nanofabrication, new sensing methods and how researchers are investigating ways to integrate biosensors within stents are highlighted. The future of implantable medical devices in the context of the emerging “Internet of Things” and how this will significantly influence future biosensor technology for future generations are also discussed.

## Graphical abstract

**The medical Internet of Things** is set to grow over the coming years. Cardiovascular disease accumulates more deaths worldwide than any other disease. Stents are one of the major treatments for cardiovascular disease and are currently going through major technological advances. These include adaptations to the fabrication materials, the addition of nanofabricated sensors, and wireless telemetry to communicate with them.

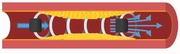

## 1 Introduction

Today, the implantable medical devices (IMDs) and technology market have expanded rapidly with the United States Office of Economics valuing the market at $400 billion in 2018.^1^ This covers a wide variety of IMDs from orthopedic to cardiovascular implants, such as stents. Stents are normally tubular, with a circular cross section, implantable vascular scaffold devices that retain their shape when deployed in a vessel. Stents have varied deployment locations from within the biliary duct to coronary artery; all use the stent to reopen blocked vessels. As such the stent market is worth $7.98 billion with 67.3% of this from coronary stents alone.^2^ This market is driven by the global epidemic of cardiovascular disease (CVD).^3^ Cardiovascular disease is in fact an umbrella term for a variety of diseases that range from myocardial infarctions to strokes to chronic kidney disease. In 2016 coronary artery disease (CAD), the formation of atherosclerotic plaques in coronary arteries, accounted for ≈9.43 million deaths worldwide making it the largest killer in the world.^3^ The American Heart Association predicts the direct and indirect costs, loss of work hours, of CVD to reach $1.1 trillion in 2035.^1^ As such the use of IMDs, specifically stents, to overcome CVD is urgent and a rapidly developing field.

**Figure 1:**
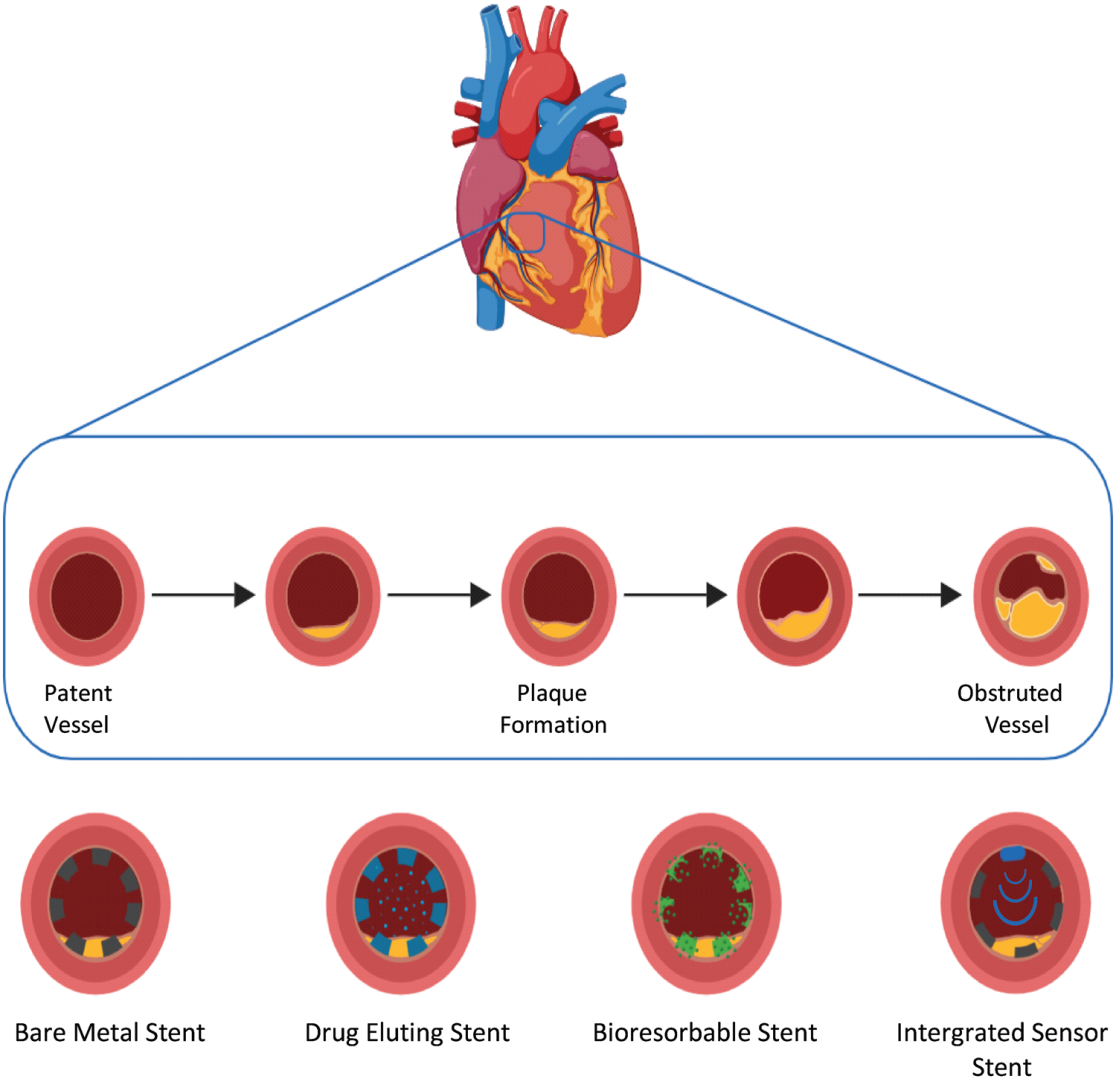
Schematic of atherosclerosis plaque formation over time and stenting devices within a coronary artery. Created with BioRender.com.

The coronary blood supply consists of a left and right‐sided system that supplies blood to the muscle of the heart. This consists of three main coronary arteries; (1) left anterior descending artery and (2) circumflex artery, and the (3) right coronary artery, which have smaller side arteries that form side branches. Like the main vessels these side branches have a reduced diameter, the further they are from their origin at the aorta until they transition into the micro vasculature. A highly established and effective tool for treating CAD is percutaneous coronary intervention (PCI). This technique involves inserting a series of catheters and guiding wires through the patients skin into their artery, either radially from the wrist's brachial artery or less commonly now the leg's femoral artery. During the procedure, the catheter is thread up through the arterial vasculature to the point of occlusive disease within the coronary blood supply of the heart. Then, a stent mounted on a thin balloon is inflated and deployed by being passed through the catheters. The balloon initially pushes back the plaque material; a product of decades long accumulated fat deposits and cellular components including smooth muscle cells, and inflammatory cells inside the vessel wall, freeing the lumen of obstruction.^4^ Then the metal stent on the balloon expands in turn with the balloon but on deflation of the balloon the stent remains in an expanded form in the vessel to stop the plaque recoiling and re‐encroaching of the plaque back into the lumen that can cause postoperative ischemic events (**Figure** **1**). The number of PCIs performed in the United Kingdom (UK) in 2015 reached 97 376 and with an ageing population the number of cases is only set to continue rising over the coming years.^5^ Due to its success, a greater number of people are surviving CAD and many of these patients live with a stent for the remainder of their lives.

PCI is highly beneficial with patients treated having improved symptoms, it has increased comfort due to the procedure being vastly less invasive than coronary artery heart bypass graft surgery, and it has significantly reduced costs and led to faster in‐hospital recovery.^6^ However, the procedure has a nemesis known as in‐stent restenosis (ISR), which is when cell overgrowth termed restenosis occurs close to or in the stents themselves. In the first generation of bare metal stents (BMS) 17%–41% of implants developed ISR.^7^ The second generation of stents had drugs coated on the stent to inhibit restenosis. These drug eluting stents (DES) are coated with antiproliferatives, a drug coating that inhibits cell growth, such as Paclitaxel or Sirolimus, or similar derivatives. Paclitaxel is a cytoskeletal drug that is an anti‐tubulin that stops microtubules formation and thus limits neointima growth.^8^ While Sirolimus is an mTOR inhibitor and thus reduces vascular smooth muscle cell proliferation.^9^ Secondary benefits of these coatings decrease ISR via the prevention of platelet aggregation, fibrin deposition, and clot formation, thus increased risk of stent thrombosis. Indeed patients are left on antiplatelet medication to prevent blood clot formation for many months after PCI.

Previous research has reported mortality from stent thrombosis occluding the vessel to be between 11% and 42% causing a type of heart attack termed ST elevation myocardial infarctions (STEMIs),^10^ whereby the ST segment of a patient's electrocardiogram is elevated due to the underpinning heart disease.^11^ However, this was reported to be reduced to 2% mortality due to STEMIs from stent thrombosis but STEMIs induced by stent thrombosis were still reported at 11%.^12^ As such the risks imposed by ISR can be deadly. Several trials have been undertaken to compare the efficacy of DES over BMS, as the review will highlight. With DES ISR was reduced to <10%.^7^ However, the odds ratio (OR) of ISR in multivessel disease increases significantly even with DES deployment in two‐vessel disease, where two out of the three coronary arteries are affected (OR: 2.922; 95% confidence interval (CI): 1.266–6.745; *p* = 0.012), or three‐vessel disease (OR: 2.574; 95% CI: 1.128–5.872; *p* = 0.025).^13^ With risks still prevalent after DES, alternate stenting devices is a field of high innovation and significant research interest.^4^

This review highlights developments in coronary artery stents by evaluating the previous bare metal stents and drug eluting stents technologies. We then assess bioresorbable stents (BRS) that are currently available, but with their own limitations. Within this, a brief overview of emerging bioresorbable stents is compared. Current research for stents and related vascular technologies is then discussed. Within this several microelectromechanical systems (MEMS) are reviewed with a vascular focus. An overview of some of the current challenges that limit these devices from being human ready vascular IMDs is finally discussed.

## 2 Established Stenting Technology

The UK National Institute for Health and Care Excellence (NICE) has suggested the use of DES for diseased coronary vessels with a diameter of <3.0 mm or with atherosclerotic lesions >15 mm in length. Posthumous analysis in the BASKET‐PROVE trial highlighted that ISR for BMS versus DES was 5.4% versus 0.76%, respectively; *p* < 0.001, with improvements at lengths and vessels outside of these.^14^ Kereiakes in a propensity matched analysis for dual antiplatelet therapy (DAPT) improvements at 12 months compared to 33 months reported major adverse cardiac and cerebrovascular events (MACCEs) and total death to be reduced in the DES at 33 months than the BMSs group (respectively, 11.4% vs 13.2%; *p* = 0.053 and 4.2% vs 5.1%; *p* = 0.16).^15^ Although not statistically significant, the total stent thrombosis was significantly reduced in DESs compared to BMS, respectively, 1.7% versus 2.6%; *p* = 0.01, with MACCE showing a noninferiority, *p* = < 0.001. Marco Valgimiglis group assessed whether potential DES recipients who were receiving similar DAPT longevities would benefit additionally from a DES over a BMS. Again, all course mortality showed no significant difference between DES and BMS, respectively, 11.1% versus 11.4%; *p* = 0.83.^16^ However, definite or probable stent thrombosis between the groups was significantly decreased, DES 2.0% versus BMS 4.1%; *p* = 0.019. This showed that an appropriate length of DAPT with DES would decrease stent thrombosis potentially improving long‐term outcomes. Sabaté in the EXAMINATION trial at 1 year showed improvements in DES over BMS for stent thrombosis and target vessel revascularization (TVR), respectively, 0.9% versus 2.5%; *p* = 0.019 and 3.7% versus 6.8%; *p* = 0.0077.^17^ However at 5 year analysis of stent thrombosis had no significant difference between DES and BMS, respectively, 2% versus 2%; *p* = 0·25; however, TVR was lower in DES compared to BMS, respectively, 7% versus 10%; *p* = 0.009.^18^ Although the stent thrombosis was similar in both stent groups, the reduction in retreating the culprit vessel notes potentially cost saving and improved patient quality of life (QOL) by reducing the costs of intervention. However, Feinberg in a Cochrane database meta‐analysis reported that DES did not significantly decrease the risks of absolute death or major adverse cardiac events (MACE) (BMS vs DES, respectively, absolute death 7.74% vs 6.97%; MACE 6.63% vs 6.36%).^19^ Feinberg also stated that the studies reviewed did not assess patient QOL and as such DES may not improve QOL long term in patients.^19^

## 3 Evaluation of Current Bioresorbable Stenting Solutions

### 3.1 Benefits and Limitations of Bioresorbable Stents

The latest advance in stenting technologies is the development of BRS that overcome many of the limitations of metal stents. BRS can be made of natural substances such as cornstarch and will naturally degrade overtime during the healing process post‐PCI. These stents have been introduced to overcome the challenges that a nondegradable BMS and DES face. These challenges include first that BMS and DES are permanent additions to the vessels that can never be removed, and they can also permanently disrupt the fine endothelial layer of the artery, disrupting the normal physiological conditions of the artery that leads to endothelial dysfunction.^20^ Second, the flow of blood through a stented artery is different to that of a nondiseased patent artery and this change in flow condition can also affect the endothelium function.^20^ Third, as these stents are permanent implants systemic antiplatelet therapy is needed for a lengthy period after implantation to prevent blood clots. A reduced conformity to the vessel in complex individuals can also lead to side branch blocking in these patients.^21^ While the long‐term polymer exposure may be related to chronic inflammation and hypersensitivity.^22^

In contrast BRS have the unique ability to overcome many of these issues. After implantation they are eventually broken down and dissolve into the blood. This characteristic overcomes the need for long‐term antiplatelet therapy, removing the risk of local hypersensitivity and chronic inflammation.^21^ Once dissolved BRS aim to leave a normal patent vessel with uninterrupted blood flow. The removal of the stent also has the potential to reinstate the vasoconstrictive properties of the vessel to sustain natural blood flow.

### 3.2 Effects of Construction Materials on Bioresorbable Stents

The magic of BRS degradation and its duration is dependent on the construction material used within the stent. The constructs can be of a polymer base or a corrosive metal that naturally degrades overtime. Polymers commonly from the monomer poly‐L‐lactide (PLLA) with derivatives of poly‐D,L‐lactide (PDLLA) or ultrahigh‐molecular‐weight PLLA are not uncommon.^23^ Polymer‐based stents with the additive of polylactic acid cross linked within them have increased longevity of stent degradation. PLLA in comparison the PDLA‐based stent can only withstand degradation longer than by up ≈20 months.^24^ However, metallic stents have an increased tensile modulus allowing for less deformity and risk of recoil with a thinner stent strut. To overcome this a polymer‐based stent needs a thicker stent strut that can therefore impede the flow of blood through the artery and instigate ISR.^24^ Polymer‐based stents also have the drawback of being radiopaque, which makes imaging of the device difficult even with markers placed on the device.^25^

Metallic constructs for BRS undergo a corrosive process in order to remove the stent from the artery. These metallic‐based stents are visible under imaging and have performed similarly to metal stents due to their similar content. However, the corrosive effect can lead to rapid degradation of the stent and as restenosis can occur up to 18 months after deployment this may be suboptimal. Magnesium succumbs to this rapid degradation while having a tensile strength that allows for minimal recoil.^26^ To increase the longevity of a magnesium‐based stent the incorporation of nicotinamide adenine dinucleotide can add protective layers to the stent.^27^ Magnesium stents offer a high tensile strength but were subject to cracking during deployment due to a low elastic strength.^28^ Manganese and tin were added to create a magnesium alloy that had decreased the rate of stent degradation while improving the mechanical properties for the stent.^29^ Manganese is of particular interest due to the inflammatory system interactions that are associated with CVD. Iron BRS were shown to have greater conformity to the vessel due to a higher elasticity modulus with no ISR noted at <18 months.^30^ In addition to this iron constructed stents have an increased longevity compared to a magnesium stent while also having greater strength allowing for slimmer stent struts.^31^, ^32^, ^33^ The addition of manganese to iron stents creates an anti‐ferromagnetic alloy, thus potentially allowing for magnetic resonance imaging compatibility.^34^ Zinc alone is not mechanically suitable for stents due to poor tensile strength and being a relatively soft metal. Alloys of zinc can utilize the malleability of the metal to produce a conforming device that in vivo studies have shown to have intermediate resistance to the corrosive process. The alloys of zinc include magnesium and manganese that have previously shown biocompatibility and efficacy for magnesium as a stent.^25^ Interestingly a zinc calcium alloy has also been investigated for future use; however, the effects of calcium release in an already diseased vessel are as yet unclear.^35^

### 3.3 Comparison of Clinical Outcomes with Bioresorbable Stents

**Figure 2:**
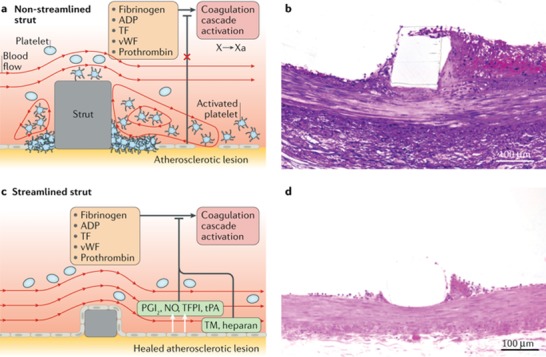
a) Schematic of a stent strut causing disruption of the laminar blood flow resulting in platelet aggregation and reduction in vasomotive signaling molecules. b) A histological cross section of a stent strut surrounded by atherosclerotic platelet legion; the blood flow here was left to right. c) Schematic of an ultrathin stent strut promoting laminar flow keeping an intact endothelial barrier and allowing vasodilation promoters to act. d) A histological cross section of an ultrathin stent strut with minimal platelet aggregation surrounding the strut. NO, nitric oxide; PGI2, prostacyclin; TF, tissue factor; TFPI, tissue factor pathway inhibitor; TM, thrombomodulin; tPA, tissue plasminogen activator; vWF, von Willebrand factor; X, coagulation factor X; and Xa, activated coagulation factor X. Reproduced with permission.^20^ Copyright 2018, Springer Nature GmbH.

Clinicians have welcomed the use of BRS to reduce ISR. However, the initial Abbot Absorb stent had a troubled beginning with late intrasegmental failure and recoil resulting in a pause in sales, although recent articles are showing acceptable and noninferior results .^20^, ^36^, ^37^ Several companies have entered the market with different designs and construction materials. One of the major findings from the failures with BRS, but also established stents, was that the stent strut size was too large and induced turbulent flow, which resulted in the activation of platelets causing coagulation (**Figure** **2**). As such, ultrathin stent, ≈100 µm, struts have been explored for all stents but also for helping improve BRS.

Results from BRS clinical trials have not had the initial significant impact that clinicians hoped BRS would bring. The BIOFLOW V trial compared drug elution polymers and stent strut thickness. They compared market leading stents such as the Orsiro BRS by Biotronik to the Xience durable polymer (DP) everolimus‐eluting stent (EES) by Abbott Vascular. Subgroup analysis found that patients who presented with acute coronary syndromes, an umbrella term for MIs and unstable angina,^11^ showed that BRS with ultrathin struts were non‐inferior to the standard DP‐EES.^38^ Target lesion failure (TLF), defined as the composite of cardiovascular death, target vessel related MI, or ischemia‐driven target lesion revascularization (TLR), in the BRS was significantly improved over the DP‐EES, respectively, 5.6% versus 11.0%; *p* = 0.023. However, TLR and total stent thrombosis showed no significant improvement in incidences, respectively, 3.5% versus 3.9%; *p* = 0.823 and 0.5% versus 1.0%; *p* = 0.601. Similarly, Byrne in the BRS Absorb stent,^39^ Abbott Vascular, when implanted for patients suffering an MI no significant reductions in future MI or TLR were noted when compared to the Xience, respectively, 1.8% versus 3.4%; *p* = 0.51 and 4.8% versus 5.7%; *p* = 0.84. Pilgrim at 5 year follow‐up of Orsiro BRS versus Xience DP‐EES showed no significant difference in TLF,^40^ TLR, or stent thrombosis; however, all cause mortality was increased in the Orsiro, respectively 14·1% versus 10·3%; *p* = 0·017. de Winter investigating in the DESSOLVE III trial of a Sirolimus‐eluting BRS,^41^ MiStent by Stentys, versus Xience again showed TLR, definite stent thrombosis and MACE to all have no significant improvements over the DP‐EES. Quantification of neointimal hyperplasia by optical coherence tomography optical coherence tomography (OCT) showed area, volume, and obstruction of neointimal hyperplasia to be reduced in the BRS over the DES, area 0·98 versus 1·31 mm^2^; *p* = 0·0022, volume 19·6 versus 32·6 mm^3^; *p* = 0·0150 and obstruction 15·0% versus 18·9%; *p* = 0·0081.^26^ In a first‐in‐human head‐to‐head comparison of two different BRS Tenekecioglu compared the novel spiral design Mirage,^42^ Manli Cardiology, against the Absorb by OCT. This showed that at 12 months the Mirage stent had a stenosis obstruction of 28.6% while the Absorb was 18.2%, *p* = 0.046, although the spiral design did not outperform the traditional design, refinement of the design may lead to future success. Gasior evaluating the ALEX,^43^ Balton, Sirimlous‐eluting BRS compared to the Xience DP‐EES reported similar results again in TVR and all stent thrombosis to previous studies after 1 year follow‐up, 5.88% versus 4.61%; *p* = 0.10 and 1.46% versus 1.21%; *p* = 0.54. Wlodarczak in an initial report of the Magmaris,^44^ Biotronik, BRS reports in hospital TLR in 1 out of the 50 patient cohort, however at 6 months no subsequent TLR or stent thrombosis. Picard produced a meta‐analysis on the most recent randomized control trails of BRS with the conclusion that BRS to be noninferior to second‐generation DES,^45^ with the major end points all being nonsignificant, TLR 1.8% versus 1.8%; *p* = 0.93, stent thrombosis 0.4% versus 0.5%; *p* = 0.85 and MACE 7.0% versus 6.2%; *p* = 0.43.

Several iterations of BRS are being researched and trailed with many concluding noninferiority to current DES. With no definitive results consistently being produced to date this new solution to atherosclerosis and ISR will continue to be developed. As such stent technology is set to continue to explore and expand the types of materials for construction, design of the stent, but also how the stent can be utilized to provide the best quality of care.

## 4 Toward an Integrated Self‐Reporting Stent Sensor

After a stent is deployed a silent wound response termed undetected ISR post PCI can have fatal consequences. It is a blockage or narrowing of the vessel at the site the stent was deployed at. It can manifest with the same deadly consequences as that of the original CAD. Early diagnosis of ISR is currently almost impossible to detect and it relies on the patient having significant chest pain or worse a second cardiac event. Those patients fortunate to have their ISR detected still rely on lifelong medication that may require subsequent PCI with additional DES deployment or drug eluting balloons. These are major undertakings on often already unwell patients and can lead to other major and costly surgical interventions such as coronary artery bypass grafting.^46^

**Figure 3:**
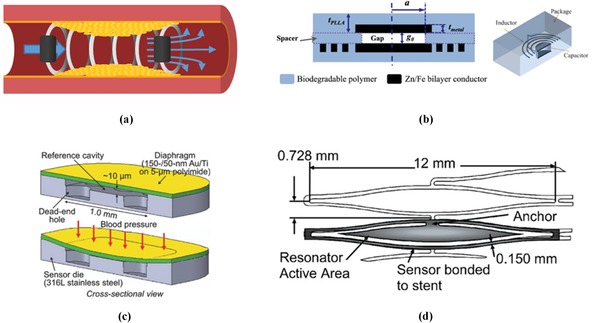
Different sensor designs. a) Schematic of stent and pressure sensors in a vessel with in‐stent restenosis. b) Cross sectional schematic and visualization of a biodegradable capacitance and resonator sensor. Adapted with permission.^49^ Copyright 2014, IEEE. c) Diaphragmatic pressure sensor with deflection of a silicon membrane when pressure is applied. Adapted under the terms of the Attribution 4.0 International (CC BY 4.0) license.^66^ Copyright 2018, The Authors, Published by Wiley‐VCH. d) Magnetoelastic resonance sensor incorporated within a stent scaffold. Adapted under the terms of the Attribution 3.0 International (CC BY 3.0) license.^56^ Copyright 2017, The Authors, Published by IOP Publishing.

Being able to remotely detect when stents are beginning to occlude would have a major impact on patient welfare, hospital inpatient costs, and decreasing the risk of major adverse events occurring unexpectedly outside the hospital environment. A solution are stents with integrated sensors that can detect growth of cells, the different cell types, and those can measure changes in flow and pressure across the lesion site. A way of achieving this within a limited space is through on stent MEMS technology. These devices will rely on Poiseuille and Bernoulli's equations for detecting differences of pressure and flow at two given points across the stent (**Figure** **3**a).^47^ As such stents integrated with sensors and using of such devices will offer an advanced solution to ISR detection and potentially wirelessly reporting changes before clinical symptoms manifest.

### 4.1 Capacitance Resonator Sensors in Stents

Another method of measuring vessel pressure wirelessly within harsh conditions was first shown by Fonseca for the use in aero plane engines operating at 0–7 bar of pressure.^48^ These sensors utilized a passive inductance and capacitance resonator. This consists of a cavity with electrodes either end that are connected to each other and acts as a variable capacitor. Please see Figure 3b for a cross sectional diagram and a constructed sensor diagram. Furthering work on passive inductance and capacitance resonator sensor Luo created a biodegradable version of the sensor,^49^ aiming for biomedical applications, with use in saline and air an operating range of 0–20 kPa was successfully measured with a sensitivity of 39 kHz kPa^−1^. The sensor was successfully stable in transmitting data for up to 86 h after immersion in saline; however, this was not submersed for the full 86 h. Chen created a thin and flexible version of the sensor and tested it in vivo using mice.^50^ This biocompatibility resulted in Park integrating the inductance sensor into a stent that was subsequently tested within a pressurized chamber to assess the sensor.^51^ An operating range of 0–230 mmHg was established with a sensitivity of 43 kHz mmHg^−1^. Furthering this work Park decreased the size of the sensor and integrated the sensor inside of the stent instead of previously outside the stent.^52^ Operating at 148 MHz with a sensing range of 0–220 mmHg when within an artificial artery, however without fluid, a linear relation of 60 kHz mmHg^−1^ was noted. Oliveira using an inductive pressure sensor upon an aortic aneurysm graft did produce fluidic‐based pressure measurements.^53^ This was performed through tubing with the stent graft over an artificial aneurysm section and a commercial pressure sensor placed before the graft to assess the pressure. The water flow was controlled by a tap that was opened and closed to mimic pulsatile flow with in the aorta. The sensor changed frequency in correspondence to the change in pressure and post analysis was able to identify frequency response for the respective pressures. Park in another study tested the sensor and antenna in vivo within a rat with successful measurement of the intravascular blood pressure; however,^54^ this was not integrated into the stent.

### 4.2 Magnoelastic Resonant Sensors in Stents

An alternate approach to detecting the change in flow was devised by Green and Gianchandani based around the resonance of magnoelastic materials,^55^ those whose magnetic susceptibility changes upon the application of mechanical stress such as Metglas and Elgiloy. The first two generations showed a proof of concept that biliary sludge could be detected as well as differentiate between healthy and nonhealthy sludge. Viswanath explored stenosis build up in peripheral artery disease using these sensors within a stent (Figure 3d).^56^ The stent and sensor were tested under flow using a peristaltic pump and successfully differentiated between diastolic and systolic pulses due to pressure changes translating into resonant difference created during systole. Following this in situ and in vivo testing was performed, which showed device interrogation at 7.5 cm was successful in transmitting data outside of the porcine mode.^57^ Further, in vivo testing of the sensor was unsuccessful due to damage of the resonators used in the sensor. The device however was biocompatible but did not show any signs of sludge formation after 1 month of implantation.^57^ The packaging of the stent was redesigned to account for the damage sustained during implantation, mainly caused due to mechanical bending during the implant of the device. The two redesigns, one stiffer and one less stiff, were both tested under stress on the bench and in situ with the most flexible reporting in situ successfully; however, no in vivo work was produced at this iteration.^58^ The in vivo work that has been performed with this sensor type has been made possible by identifying the use of peripheral stenting devices and utilizing the larger scale of these stents to allow attachment of sensors with greater ease.

### 4.3 Silicon Diaphragm Pressure Sensors in Stents

Due to the common metallic nature of stents the idea of incorporating an antenna into the stent scaffold was proposed by Takahata.^59^ The stent antenna, termed “stentenna,” used micro machined foil, which was attached to a silicon diaphragm pressure sensor. This inductive coupled stentenna successfully assessed pressure under flow in a mock artery. Mohammadi built upon this work coating the stents in copper and gold in order to improve the inductance of the antenna.^60^ On coating the helical stentenna and applying a frequency of 150 MHz the quality factor, a measure of resonator characteristics, increased to 35 for copper and to 31 for gold from a starting point of 5. The stainless steel stent peaked at 5 when a 150 MHz resonant frequency was applied. Thin film diaphragmatic transducers (Figure 3c) were investigated for increased pressure sensitivity. These sensors had a linear relation between capacitance increase and pressure increase while having a response time of 0.43 ms under dynamic flow.^61^ Thus, suitability for sensing beat to beat pressure would be possible at high human heart rates. Chen coated the gold stentenna with parylene C to insulate the electronics of the stent and prevent their degradation;^62^ parylene was chosen due to its biocompatibility.^63^ In vitro flow modeling of the parylene C coated stent showed a frequency response from 0 to 78 mmHg over an increasing flow rate of 0–900 mL min^−1^. With intracoronary flow in severe coronary artery stenosis measured at a mean of 2.54 ± 0.55 mL s^−1^ and stenosed vessels at a mean of 4.81 ± 1.95 mL s^−1^, this varying flow rate represents a working physiological range.^64^ Although the sensor was only half as sensitive during the flow experiments compared to stationary experiments, this did identify that the sensor was sensitive to flow rates, an important monitoring option. The stent was successfully integrated into a commercial graft and deployed in swine and confirmed through angiography. After initial pressure recordings the stent stopped transmitting which they speculated to be caused by an electrical fault due to mechanical bending during the procedure or graft clotting. To improve the robustness of the sensor under the crimping of the stent to the balloon and mechanical bending encountered under insertion Chen introduced laser microwelding of a redesigned sensor to the stentenna.^65^ This ongoing research has cumulated with the recent publication of Chen where a stent was crimped to a balloon at >100 Newton's and was inflated within a graft.^66^ Successful wireless interrogation through the graft allowed for data transfer until graft occlusion due to thrombosis caused by repeated compression and relaxation of the graft in order to vary pressure. The diaphragmatic sensor showed sensitivity within the required limits of the systemic and coronary circulation systems. The stentenna design reduces the electrical componentry through the novel integration to the stent scaffold and as such has the potential to decrease the electronic footprint a telemetric stent would have within a vessel.

### 4.4 Biosensors for Neurological Stents

Although this review focuses on CAD atherosclerosis stents are also clinically used for diseases found in other vessels and are treated in a similar methodology. These stents too can develop ISR. The use of stents with sensors within carotid arteries and cerebrovasculature of the brain therefore represents a deployment area opportunity and has also been pursued. Brox using the stentenna investigated the depths of transmission in which the signal could be detected.^67^ When immersed in saline the stent was not detected at a distance 12.5 mm from the receiver, thus not suitable for deployment within the chest cavity. More suitable areas of deployment were identified in superficial areas like the carotid artery. Chen produced a highly flexible mesh, stretching increase of >500% in the radial axis,^68^ thus being robust enough for applications in a flow diverter stent for the treatment of cerebral aneurysms. This mesh was then integrated with nanofabricated capacitance flow sensors.^69^ Flow was detected for a maximum of 10 min due to the electrodes dissolution, gold fabricated electrodes outperformed the longevity of Nitinol and magnesium constructed electrodes. Work within the endovascular of the cerebral cortex has produced a deployable electrode array across the motor cortex of ewes within a Nitinol‐based stent.^70^ Previous investigations of these sensors within this design noted that the wireless telemetry was ultimately the limiting factor for device progression.^71^, ^72^ Importantly, assessment of electrode integration into the stent design did not limit flow or induce malignancies.^71^, ^72^

### 4.5 Complementary Metal–Oxide–Semiconductors within Stents

**Figure 4:**
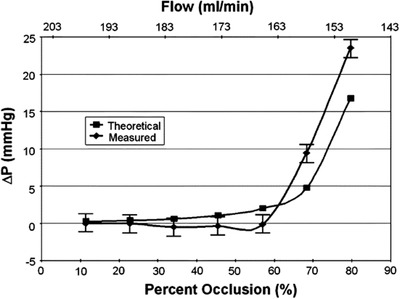
Graphical representation of flow rate and pressure changes caused by varying degrees of occlusion. Theoretical curve with a comparison to a measured curve by a BiCMOS. Reproduced with permission.^73^ Copyright 2006, IEEE.

DeHennis and Wise showed that flow and pressure were feasible detections for an implantable,^73^ wireless and battery‐free device. A description between the degree of occlusion to the changes in flow and pressure was also presented in their paper (**Figure** **4**). This was achieved through a BiCMOS, the combination of bipolar (Bi) junction transistors and complementary metal–oxide–semiconductor (CMOS) transistors. A CMOS allows for high density transistors and low power consumption, thus a power but efficient sensor. However, CMOS transistors are susceptible to noise more so than BiCMOSs. A BiCMOS integrate the low operational voltage of bipolar junctional transistors and their low input and output impedance with the logic benefits of a CMOS. Although this increased the cost of a device and the complexity of the device is increased, the result is a highly sensitive, robust, and efficient device is produced. This resulted in the detection of a reduction in flow of 13%, which is the equivalence of a 3 mmHg pressure drop. Furthering work on CMOS embedded stents Chow produced a high sensitivity sensor,^74^ 0.5 mmHg with an average error of 1.268 mmHg, and tested it in vitro over a range of pressure 0–50 mmHg. The stent itself was used as the antenna for the device and resulted in a transmission capability of 10 cm to 1 m when implanted in vivo at 3.5 cm in swine. These results within stents show the high performance^75^, ^76^ and accuracy that CMOS‐based MEMS are recognized. Further investigations into CMOS‐based stent sensors are likely due to their low production costs and probable further size reductions.

### 4.6 Sensors with Stenting Applications

The reviewed MEMS have been integrated into stenting devices for use in varied sites from the biliary duct to cerebral veins. Novel and emerging sensors are yet to be integrated into stents but potential integration would be highly beneficial. Pulse wave velocity (PWV) is the measurement of how the blood pressure pulse travels through the blood vessels to assess the elasticity of the vessel. This measure could therefore be used to detect stiffening, an important determinant of hypertension.^77^ A novel biodegradable sensor based on poly(glycerol sebacate) was used to measure pulse wave velocity in vitro. The multielectrode array had high sensitivity with capacitive changes unique when a bee (22.5 mg) and a grain of rice (21.8 mg) were measured.^78^ The authors propose a cardiac patch with the sensor built into it; however, another use due to the high sensitivity and flexibility may be within a coronary stent for pressure measurements.

Attaching sensors to the outside of a graft was shown by Milner using a pressure sensor and aortic aneurysm graft.^79^ As such many of the sensors discussed have also used and been in used in grafts. A novel approach potentially for graft occlusion detection is through Doppler ultrasonography.^80^ A wireless Doppler probe within a cuff is attached to the artery and the velocity of blood can be detected, which in turn allows for pressure and also the detection of occlusion.^81^, ^82^ Multiple sensors outside of a graft could allow for a Doppler image of the whole grafted vessel that is consistent with the Doppler flow images vascular sonographers are familiar with. Another alternate measurement system though hydrostatic pressure measurements made possible by a microbubble‐based MEMS showed that impedance changes correlated to pressure.

Alternatively, molecular markers of ISR could be detected at a cellular level using protein molecular sensors. Several biosensors that investigate the proliferation the of cells and also the biochemical changes that this causes are in development.^83^ Electrical impedance spectroscopy has the potential to accommodate the detection of cellular proliferation with endothelial cells successfully detected.^84^, ^85^ These sensors offer the ability to characterize cells and clots with current uses in catheters and a stent seeking clinical approval.^86^, ^87^, ^88^ Alternatively, endothelial dysfunction and vascular remodeling biochemical markers associated with ISR are another possible avenue. This can include inflammatory markers such as TNF or other markers like fibrin and nitric oxide.^88^ Investigations into sensors for the detection of these markers through impedance spectroscopy are promising, especially with oncology, however are not yet stent based.^89^ The use of impedance‐based sensors due to their miniaturization capabilities, accuracy, and multiple detection capabilities could be a powerful tool for future vascular applications.

## 5 Future Considerations

One of the key enabling technologies for IMDs is the Internet of Things (IoT), specifically the medical IoT. In a Forbes article the global value of the medical IoT alone was estimated to be on a trajectory to reach $136.8 billion by 2021.^90^ The IoT is the name given to the network of devices or “things” that have the ability to communicate with one and other over wireless networks and then report data to a cloud‐based server. This allows the user access to these “things” wherever and whenever they require it, in the case of medical IoT, for example, this could be IMDs or ward‐based monitoring systems. On top of this the cloud‐based storage and communication of these “things” allows for “big data,” large volumes of specific and unspecific personal data, acquisition to establish trends and predictions, for example, within the hospital and community care.^91^, ^92^ The impact of these devices on health care is becoming and will furthermore become the standardized norm adding to the decentralized health‐care model that the smart city will enable and drive forward.

Modern connected medical devices allow physicians 24 h access to a vast array of monitoring options within a hospital and with enabled IMDs allowing remote monitoring of the patient through the IoT. With the IoT driving the future of IMDs the accessibility of wireless devices has become a major security concern.^93^ Several methods have been described detailing the architectures of how this can be achieved. Limiting the access to approved individuals within health‐care system is one option while using dual authentication through a patients internal electrocardiogram and their finger print to authenticate access to patients data has also been put forward.^94^, ^95^ Constant wireless communication carries the risk of malicious attack, interference with other wireless devices, and an increased battery drain.^96^, ^97^ With the privacy of personal data a hot topic securing the device data and network needs is an important consideration that should not be underestimated from the first prototype all the way through to the finished commercial entity in order to reach regulatory approval.

The average time for devices to reach regulatory approval is shorter than medicines but on average it still takes 3 to 7 years and with cost for drugs spiraling to over $1 billion it is also expensive.^98^ Devices are classified as under the United States Food and Drug Administration (FDA) system have three classes. A class I device has low risk of illness or injury class II moderate risk and Class III a device that sustains the life or could impose high risk.^99^ The FDA automatically classes any device as class III unless an exemption is granted for approval to enter at a lower grade which can be achieved through showing its equivalency to existing devices.^100^ As such modifying an existing approved IMD may facilitate approval, than for an entirely novel device. A number of devices that enter health care use this equivalency to get to market.^101^ In addition to the FDA approval quality assurance in the form of ISO13485 will need to be achieved prior to application of the standardizing agency, this is normal 9–12 months before market release but should be considered from the prototyping stage.^102^

The full integration of the stentenna is an example of adapting current working designs for treatment and increasing their functionality to prepare them for future uses. The use of the stent as an antenna for wireless power transfer has also been investigated mathematically and in vitro.^103^ The importance of powering IMDs wirelessly revolves around the hazards associated with implanting chemical batteries within the body with size upon a stent also a limitation. Active devices, those powered by a battery, are at risk of leakage, corrosion, and production of excess heat.^104^ Transferring the power wirelessly to a stent or other IMD would therefore eliminate the battery and reduce these risks. STENTag uses radio frequency identification (RFID) to interrogate a passive sensing device, without an implantable battery, for proposed carotid artery stents.^105^ In vitro testing within a submerged phantom vessel allowed detection of varying stenosis using this passive technology which compared to predetermined computational modeling. The use of RFID integration into a safe and potentially effective powering option however the superficial implantation in the carotid artery may not be as successful in arteries with thicker tissue layers and bone obstruction. Wireless power transfer to an active device offers another solution for powering IMDs.^106^ Several other investigations into powering IMDs are suggested including biofuel cells and piezoelectric muscle contraction utilization.^107^, ^108^, ^109^, ^110^, ^111^ A novel approach using in vivo work into biodegradable batteries has been performed and would therefore remove the risk of chemical leakage and lends itself to the biodegradable stents currently being used.^112^ As such, innovative passive and active powering methods are set to continue to be developed and utilized for IMDs.

The BRS is set to continue to be researched and as the IoT of things is set to grow the importance of biodegradable sensors is noted by many. Investigations into implantable biodegradable sensors for IMDs are unsurprisingly similar to the materials used in BRS. Hwang produced sensors based on poly lactic acid (PLA),^113^ which produces the co polymers of PLLA and PDLLA. The series of experiments showed that PLA derivatives with the use of various construction steps using of silicon electrodes and magnesium interconnects could produce a biodegradable CMOS.^113^ Alternatively molybdenum disulfide can be used due to its biocompatibility and the ease at which established manufacturing techniques can be used to create the components.^114^ Pressure, strain, and temperature were all able to be detected in vivo via the sensor. Previous to this a biodegradable radiofrequency antenna made from magnesium had been produced and successfully powered a light emitting diode.^115^ Within the review pressure sensors were reviewed with biodegradable sensors being studied,^78^ furthering this a biodegradable pressure and strain gauge has been devolved for orthopedic use; however, the use of a strain gauge within a graft is a possible advantage in aiding PWV.^116^

## 6 Conclusion

The use of sensors for monitoring internal conditions within a vessel is now feasible. As a direct measurement of in stent restenosis the area is focused on pressure due to the changes that can occur with plaque formation, which is similar to pressure studies interventionist cardiologist would employ currently. The use of these sensors peripherally has also been addressed, again with a pressure orientation, which would fall in line with current measurement methods but also allow for chronic measurement of a patient's blood pressure. This aligns neatly with the Internet of Things in allowing constant patient monitoring and therefore enabling big data techniques to improve the patients future health predictions based on their current status. The integration of all necessary components for a sensor, stent, and wireless communication system remains a major limiting factor. With CVD a major threat and more procedures of PCI with a stent deployment being carried out yearly the use of sensors to monitor the vessel post procedure is likely to be the next evolution of the stent.

## Conflict of Interest

The authors declare no conflict of interest.
